# The Use of CA-125 KELIM to Identify Which Patients Can Achieve Complete Cytoreduction after Neoadjuvant Chemotherapy in High-Grade Serous Advanced Ovarian Cancer

**DOI:** 10.3390/cancers16071266

**Published:** 2024-03-24

**Authors:** Dimitrios Zouzoulas, Dimitrios Tsolakidis, Panagiotis Tzitzis, Iliana Sofianou, Kimon Chatzistamatiou, Vasilis Theodoulidis, Maria Topalidou, Eleni Timotheadou, Grigoris Grimbizis

**Affiliations:** 1 1st Department of Obstetrics & Gynecology, “Papageorgiou” Hospital, Aristotle University of Thessaloniki, 56403 Thessaloniki, Greece; 2 Radiotherapy Department, “Papageorgiou” Hospital, 56403 Thessaloniki, Greece; 3 Department of Oncology, “Papageorgiou” Hospital, Aristotle University of Thessaloniki, 56403 Thessaloniki, Greece

**Keywords:** neoadjuvant therapy, ovarian cancer, KELIM score

## Abstract

**Simple Summary:**

Residual disease after cytoreduction in advanced ovarian cancer still remains the most important prognostic factor. Patients with high tumor load, especially in the upper abdomen, that cannot be completely debulked in the primary setting are offered neoadjuvant chemotherapy and reassessed after 3–4 cycles for interval debulking surgery. Imaging is the main tool for the decision if these patients can achieve complete cytoreduction. However, even with the use of the Response Evaluation Criteria in Solid Tumors (RECIST), conventional imaging has poor results in identifying which patients have unresectable disease. Therefore, the KELIM score appears as a new promising prognostic factor in the neoadjuvant setting. The aim of this study is to establish KELIM as prognostic factor for residual disease after interval debulking surgery and to urge the research community to plan future randomized control trials in order to add the KELIM score in future guidelines.

**Abstract:**

(1) Background: Neoadjuvant chemotherapy followed by interval debulking surgery is used in the treatment of advanced ovarian cancer. However, no tool can safely predict if complete cytoreduction after 3–4 cycles can be achieved. This study aims to investigate if the KELIM score can be a triage tool in the identification of patients that will be ideal candidates for interval debulking surgery (IDS). (2) Methods: We retrospectively analyzed the records of patients with high-grade serous advanced ovarian cancer that were treated in the 1st Department of Obstetrics–Gynecology, 2012–2022, with neoadjuvant chemotherapy followed by IDS. Patient characteristics, oncological outcome and follow-up information were collected. The primary outcome was the association of the KELIM score with residual disease. (3) Results: 83 patients were categorized into two groups: Group A (51 patients) with favorable (≥1) and Group B (32 patients) with unfavorable (<1) KELIM scores. A statistically significant correlation between KELIM and residual disease (*p* < 0.05) exists, showing that patients with a favorable KELIM score can achieve a complete IDS. Furthermore, there was a statistically significant difference in overall survival (*p* = 0.017), but no difference was observed in progression-free survival (*p* = 0.13); (4) Conclusions: KELIM seems to safely triage patients after neoadjuvant chemotherapy and decide who will benefit from IDS.

## 1. Introduction

The standard treatment plan for patients with advanced ovarian cancer is debulking surgery and platinum-based chemotherapy. Primary debulking surgery is the preferred upfront treatment [1], but patients with high tumor load, especially in the upper abdomen, that cannot be completely debulked in the primary setting could benefit by the administration of neoadjuvant chemotherapy followed by interval debulking surgery. Even though three randomized control trials [2,3,4] showed that neoadjuvant chemotherapy followed by interval debulking is not inferior to primary debulking surgery followed by adjuvant chemotherapy, the debate is still ongoing and the results of the TRUST trial are awaited [5].

The success of this treatment plan depends on tumor chemosensitivity [6] and the ability to achieve complete interval debulking surgery, because residual disease after cytoreduction remains the most important prognostic factor [7,8,9,10]. Imaging is the main tool to assess tumor resectability in order to achieve complete cytoreduction. However, even with the use of the Response Evaluation Criteria in Solid Tumors (RECIST) [11], conventional imaging has poor results in identifying which patients will have unresectable disease [12]. Furthermore, laparoscopy after neoadjuvant chemotherapy has been proposed as a triage tool, but its value remains controversial in the prediction of complete cytoreduction [13]. On the other hand, 15–20% of patients with advanced ovarian cancer will be poor responders to chemotherapy [14], so there is the need for accurate non-invasive chemosensitivity predictors to guide treatment decisions in the first-line setting, which is acknowledged by ESGO and ESMO [15].

Monitoring of CA-125 decline during chemotherapy as a predictor of treatment response [16] and as a way to overcome imaging limitations, has been one of the main points of research in ovarian cancer patients [17,18]. Many types of CA-125 measurements have been proposed in the recent years. Firstly, the CA-125 nadir level, half-life value and time to nadir have been proposed [19] and secondly, the Gynecologic Cancer InterGroup (GCIG) defined CA125 based response as a 50% reduction in the CA125 level maintained for at least 28 days [11]. However, both have controversial results and failed to accurately predict chemosensitivity [20]. Recently, the ELIMination rate constant K (KELIM), a modeled kinetic parameter based on CA-125 measurements during the first 100 days of systemic therapy (adjuvant of neoadjuvant chemotherapy), has emerged as a valuable predictor [21]. It is a mathematical modeling method based not on absolute values of the biomarker, but on the longitudinal kinetics (CA-125 elimination) during treatment, completely independent of renal function. Two recent meta-analyses have shown that it is an independent prognostic biomarker for survival outcomes and that can predict chemosensitivity [16,22]. The higher the KELIM score, the faster the CA-125 elimination, the higher the chemosensitivity and the better the prognosis [22]. The aim of this study is to establish KELIM as prognostic factor for residual disease after interval debulking surgery.

## 2. Materials and Methods

### 2.1. Study Characteristics

Retrospective analysis of women with newly diagnosed advanced ovarian cancer, who were treated in the 1st Department of Obstetrics & Gynecology, AUTh, “Papageorgiou” General Hospital, from 1 January 2012 until 31 December 2022 and identification of those that underwent platinum-based neoadjuvant chemotherapy followed by interval debulking surgery. The total number of patients in this period of time was 324. A written approval was received from the Institutional Review Board.

### 2.2. Patients

Inclusion criteria:

- Newly diagnosed advanced ovarian cancer;
- High-grade serous histological type.

Exclusion criteria:

- Primary debulking surgery;
- Missing important registry data of CA-125 values to calculate KELIM.

As a result, 196 out of the 324 women with ovarian cancer were excluded due to primary debulking surgery or as a recurrence of ovarian cancer. Moreover, 45 women were excluded, because they were missing important registry data of CA-125 values to calculate KELIM. Hence, finally 83 women with high-grade serous advanced ovarian cancer were identified as eligible for further analysis.

### 2.3. Data Collection

Data were collected during a period of one month. Our Gynecological–Oncology Unit has an online registry system with all the relevant data of the patient’s medical records. In order to avoid inconsistencies among different dates of data collection, a uniform data collection sheet (excel file) was used, during the retrospective mining of the patient’s medical records. The data sheet included the following information:

- Patient’s identifiers:
  ∘ Name
  ∘ Hospital identification number
- Patient’s age
- Body Mass Index (BMI)
- Charlson Comorbidity Index (CCI) [23]
- CA-125 serial values during neoadjuvant chemotherapy
- KELIM Score
- Intensive Care Unit (ICU) admission
- Clavien-Dindo classification for post-operative complications [24]
- Hospital stay
- Residual disease after debulking surgery with Peritoneal Cancer Index (PCI score)
- Time related data:
  ∘ Date of diagnosis
  ∘ Date of recurrence or disease progression
  ∘ Date of last follow-up or death

The KELIM score was measured in the neoadjuvant setting with the available online tool [25]. The KELIM score was analyzed as a continuous and as a binary index test with the cut-off point of 1 or greater (≥1) for favorable result. The dates of every cycle of chemotherapy were entered and also the relevant values of CA-125 within the first 100 days from the start of neoadjuvant chemotherapy. Preferably the CA-125 values before cycles 2, 3 and 4 were used to calculate KELIM score, but if one was missing, the CA-125 value prior to the 1st cycle of chemotherapy (within 7 days from neoadjuvant chemotherapy start) was taken into account, which was the case for only seven patients.

### 2.4. Statistical Analysis

In the statistical analysis, the baseline characteristics of the patients who participated in the study were calculated. There was no case of missing data. Continuous variables are demonstrated as means with standard deviation (SD) while categorical variables with frequencies and percentages (%). Univariable and multivariable analysis was performed. Progression-free (PFS) and overall survival (OS) analyses were performed using the Kaplan–Meier curves and the groups were compared using the log-rank test and Cox regression. PFS was defined as the time interval between date of diagnosis and date of first recurrence or disease progression, while OS as the time interval from diagnosis to the date of death or last follow-up. A test of normality was conducted using Shapiro–Wilk and Kolmogorov–Smirnov tests. All reported *p*-values were two-tailed at a 5% significance level. We analyzed data using R statistical software (R Project for Statistical Computing), version 4.3.0.

## 3. Results

This retrospective cohort study included 83 patients with high-grade serous advanced ovarian cancer that were offered platinum-based neoadjuvant chemotherapy. Patients’ characteristics are outlined in Table 1. The mean age of the women at the time of the diagnosis was 62 years old, while the mean BMI was 28 kg/m^2^, meaning that most women were overweight. Furthermore, concerning the performance status of our patients, almost half of them (47%) had mild to moderate comorbidities, which was measured by the Charlson Comorbidities Index. All patients received 3 or 4 cycles of neoadjuvant chemotherapy and the majority of them (76%) had FIGO Stage III disease. The KELIM score was calculated with serial values of CA-125 during neoadjuvant chemotherapy and nearly two thirds of the patients (61.5%) had a favorable KELIM score (≥1). Moreover, regarding postoperative complications, the median value of the Clavien–Dindo classification was 22.6, with an IQR of 12.2–32. Only 14 (16.9%) patients required ICU admission and the median hospital stay was 8 days, with an IQR of 6.5–9. The PCI score of the patients was calculated at the start and at the end of cytoreduction. Residual disease (RD), which was the primary endpoint of our study, was calculated using the PCI score [26,27]. RD was present in 25 (30%) patients, meaning that 58 (69.9%) patients underwent a complete interval debulking surgery. Further analyzing the patients with residual disease, 14/83 patients (17.1%) had an optimal debulking surgery (residual disease < 1 cm), leaving only 13.2% of the patients that underwent a suboptimal debulking surgery (residual disease ≥ 1 cm).

The primary outcome of our study was the association of the KELIM score with residual disease after neoadjuvant chemotherapy followed by interval debulking surgery. Considering the KELIM score as a binary value and the cut-off point at 1, two groups of patients were identified: Group A, with a favorable KELIM score (≥1), which included 51 (61.4%) patients and Group B with unfavorable KELIM score (<1), which included 32 (38.6%) patients. There was no statistically significant difference between the two groups concerning age, BMI, comorbidities, FIGO Stage, chemotherapy cycles, postoperative complications, hospital stay and ICU admission. On the other hand, a strong association between the KELIM score and residual disease was found (*p*-value < 0.05) in the univariate and multivariate analysis, identifying patients with a favorable KELIM score as ideal candidates to achieve complete debulking surgery in the neoadjuvant setting. However, there were eight patients with unfavorable KELIM scores (<1) that were completely debulked and only one case of a favorable KELIM score (≥1) that resulted to an optimal debulking surgery (residual disease < 1 cm), due to milliary disease in the small bowel. The aforementioned data are detailed presented in Table 2 and Table 3.

**Figure 1:**
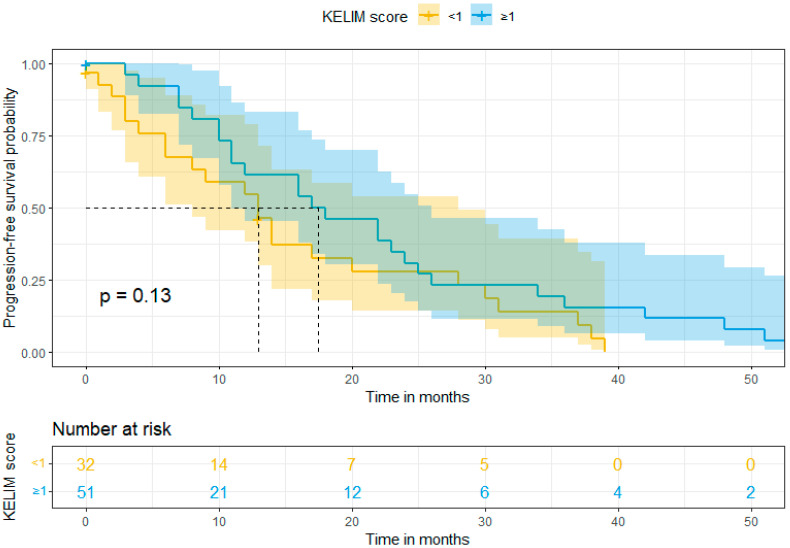
Progression-free survival (Kaplan–Meier curve).

**Figure 2:**
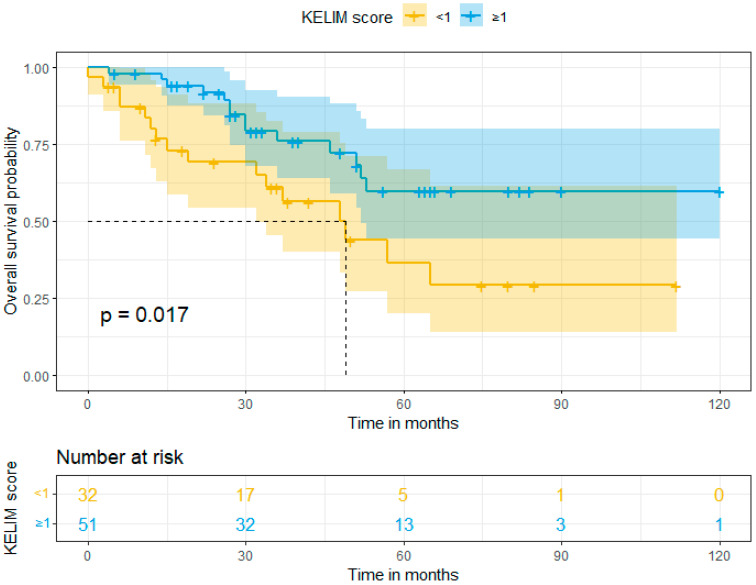
Overall survival (Kaplan–Meier curve).

In our study, the mean follow-up was 39 months (range: 0–120). Survival rates were calculated using Kaplan–Meier curves. The median overall survival (OS) in Group A (favorable KELIM score) and Group B (unfavorable KELIM score) was >120 and 48 months, respectively. On the other hand, the median progression-free survival (PFS) was 18 months and 13 months, respectively. By using log-rank tests, no statistically significant difference was found in the PFS (*p* = 0.13), but a statistically significant difference was observed in OS (*p* = 0.017) between the two groups in favor of Group A. These results are shown in Figure 1 and Figure 2. Moreover, after performing univariate and multivariate analysis by using Cox regression, the association between KELIM score and overall survival became not statistically significant in the multivariate analysis. Table 4 and Table 5 present the aforementioned statistical analysis.

## 4. Discussion

The primary objective of our study was to investigate if the KELIM score can predict residual disease after neoadjuvant chemotherapy followed by interval debulking surgery. There is a need for a triage tool that can identify the patients that will benefit from a debulking surgery, but also the patients that will need further cycles of neoadjuvant chemotherapy and a delayed interval debulking surgery. The association between the KELIM score and survival rates (progression-free and overall survival) was studied as a secondary result, in order to establish KELIM score as a prognostic factor. We designed a retrospective study to test if the KELIM score is an independent index in the neoadjuvant setting, because these patients have primary unresectable high tumor load and are associated with a worse prognosis.

Eighty-three patients were included in the study and were divided into two groups based on the KELIM score, which was measured with at least three serial CA-125 values at specific time intervals in association with the exact date of neoadjuvant chemotherapy cycles. Group A included 51 patients with a favorable KELIM score (≥1) and Group B 32 patients with an unfavorable KELIM score (<1). A statistically significant association was found between the KELIM score and residual disease after interval debulking surgery, showing that patients with a favorable KELIM score are ideal candidates for successful complete interval debulking surgery.

However, there were some cases in which the KELIM score did not correctly predict the residual disease in the neoadjuvant setting. Specifically, eight patients with an unfavorable KELIM score ended up being completely debulked. After independently reviewing the PCI and the Alleti score of all eight patients, we found a high tumor load even after neoadjuvant chemotherapy that required expert surgical skills, which included upper abdomen radical resections and en block Hudson pelvic procedures. On the other hand, there was only one case of a favorable KELIM score that did not result in complete, but rather in an optimal debulking surgery (residual disease < 1 cm), due to milliary disease on the serosa and mesentery of the small bowel. It is important to mention that this patient was assessed and declared unresectable, prior to neoadjuvant chemotherapy, with imaging and not with diagnostic laparoscopy.

Furthermore, our study failed to demonstrate a statistically significant difference in progression-free survival, even though patients with an unfavorable KELIM score showed a worse survival rate. This might be explained by the fact that the eight aforementioned patients with a KELIM score < 1 were debulked to zero residual disease, because they were treated in an ESGO-certified center for advanced ovarian cancer and they were offered the maximum surgical effort and expertise. In contrary, a statistically significant difference was found in overall survival, where patients with a favorable KELIM score did not yet reach the median overall survival in the follow-up period (median just over 3 years). However, after analysis for possible confounding factors in the multivariate analysis, no association between KELIM score and death of disease was found, mainly due to the relevant small population of the study.

To our knowledge, there are only a few studies in the literature that investigate the role of the KELIM score in the neoadjuvant setting for the treatment of advanced ovarian cancer. Two recent meta-analyses [16,22] that studied the KELIM score in the adjuvant, neoadjuvant and recurrent setting showed that is an independent prognostic biomarker for progression-free and overall survival, which can predict chemosensitivity.

The first study published in 2017 by Ducoulombier et al. [28] was a retrospective cohort of 54 patients, which showed that the KELIM score was an independent predictor for optimal cytoreduction after neoadjuvant chemotherapy. This result is in accordance with our findings, but with a key difference in the main endpoint, which was complete and not optimal debulking surgery. The highest quality data came from a post hoc study [29], which included 134 patients from the randomized phase II CHIVA trial and showed that KELIM score is an independent prognostic factor of complete interval debulking surgery and a favorable KELIM score is strongly associated with a better progression-free and overall survival. These results were verified by two studies that were recently published, a retrospective cohort of 232 patients [30] and a national cancer registry database of 1.582 patients [31], and are in agreement with our findings, except from progression-free survival. This may be due to the fact that 8 patients with an unfavorable KELIM score underwent extensive radical interval debulking surgery in order to achieve zero residual disease, which is an independent prognostic factor for survival outcomes. On the other hand, another retrospective cohort of 217 patients [32] showed no association of the KELIM score and no gross residual disease after interval debulking surgery, but confirmed the association between favorable KELIM scores and better survival rates.

Our study was conducted in a university, tertiary ESGO-certified for advanced ovarian cancer surgery center. All the required parameters were collected from an online system, therefore minimizing the percentage of missing important data and all CA-125 values were measured in the same laboratory of our hospital. Furthermore, our study has the longest median follow-up period and the highest complete resection rate, almost 70%, while in most studies it was less than 50%, which is a benchmark in the ESGO quality indicator for advanced ovarian cancer surgery. In contrast, the main limitation of our study is the low number of the population included in the final analysis and its retrospective nature.

Future large prospective studies are needed in order to establish the KELIM score as a prognostic factor in the neoadjuvant setting, because it is a cheap, easily accessible and reproductible tool.

## 5. Conclusions

KELIM seems to be a valuable triage tool to safely choose patients after neoadjuvant chemotherapy and decide who will benefit from interval debulking surgery. On the other hand, patients with an unfavorable KELIM score should be referred for diagnostic laparoscopy to assess tumor resectability in order to avoid incomplete cytoreductions.

## Figures and Tables

**Table 1:** Patient characteristics.

Characteristics		Number of Patients (N)	Percentage (%)
Age (years)		mean: 62	SD: 12.3
BMI		mean: 28	SD: 5.9
CCI		median: 2	IQR: 1–4
	0–2	44	53
	3–4	25	30.1
	≥5	14	16.9
FIGO Stage			
	III	63	75.9
	IV	20	24.1
Chemotherapy cycles			
	3	73	88
	4	10	12
KELIM score			
	<1	32	38.5
	≥1	51	61.5
Clavien-Dindo classfication		median: 22.6	IQR: 12.2–32
ICU Admission		14	16.9
Hospital Stay (days)		median: 8	IQR: 6.5–9
Residual disease (cm)			
	0	58	69.9
	<1	14	16.9
	≥1	11	13.2

BMI: Body Mass Index, CCI: Charlson Comorbidity Index, ICU: Intensive Care Unit.

**Table 2:** Comparison based on KELIM score.

Characteristics		Group A (≥1) 51 (61.4%)	Group B (<1) 32 (38.6%)	*p*-Value
Age (years) mean (SD)		61.2 (11.6)	63.4 (13.3)	0.436
BMI (kg/m^2^) mean (SD)		28.9 (5.5)	26.9 (6.4)	0.277
CCI median (IQR)		2 (1–4)	2.5 (1–4)	0.383
Clavien–Dindo classification median (IQR)		21.8 (12.2–30.2)	23.4 (15–33.7)	0.310
FIGO Stage n (%)				0.796
	III	38 (74.5%)	25 (78.1%)	
	IV	13 (25.5%)	7 (21.9%)	
Chemotherapy cycles n (%)				0.498
	3	46 (90.2%)	27 (84.4%)	
	4	5 (9.8%)	5 (15.6%)	
ICU admission n (%)				0.717
	Yes	8 (15.7%)	6 (18.8%)	
	No	43 (84.3%)	26 (81.3%)	
Hospital stay (days) median (IQR)		8 (6.5–9)	7.5 (6.5–9)	0.815
Residual disease (cm)				**<0.001**
	0	50 (98%)	8 (25%)	
	<1 or ≥1	1 (2%)	24 (75%)	

BMI: Body Mass Index, CCI: Charlson Comorbidity Index, ICU: Intensive Care Unit.

**Table 3:** Logistic regression for residual disease.

Characteristics		Univariable			Multivariable		
		OR	95% CI	*p*-Value	OR	95% CI	*p*-Value
Age (years)		1.01	0.97, 1.05	0.649			
BMI (kg/m^2^)		0.94	0.84, 1.05	0.237			
CCI		1.18	0.95, 1.47	**0.140**	1.11	0.71, 1.71	0.654
Clavien–Dindo classification		1.00	0.97, 1.03	0.949			
FIGO Stage							
	III	7.89	2.59, 24.03	**<0.001**	12.86	0.67, 246.59	0.090
	IV	1	1	1	1	1	1
Chemotherapy cycles							
	3	0.38	1.00, 1.44	**0.155**	5.66	0.19, 169.03	0.317
	4	1	1	1	1	1	1
ICU admission							
	Yes	1.36	0.40, 4.57	0.618			
	No	1	1	1			
KELIM score							
	<1	1	1	1	1	1	1
	≥1	0.007	0.001, 0.056	**<0.001**	0.007	0.001, 0.071	**<0.001**
Hospital stay (days)		0.98	0.86, 1.12	0.774			

BMI: Body Mass Index, CCI: Charlson Comorbidity Index, ICU: Intensive Care Unit.

**Table 4:** Cox regression for recurrence or disease progression.

Characteristics		Univariable			Multivariable		
		OR	95% CI	*p*-Value	OR	95% CI	*p*-Value
Age (years)		1.00	0.98, 1.03	0.860			
BMI (kg/m^2^)		1.05	0.97, 1.13	0.242			
CCI		1.12	0.97, 1.29	**0.112**	1.06	0.91, 1.23	0.488
Clavien–Dindo classification		1.01	0.99, 1.03	0.395			
FIGO Stage							
	III	2.31	1.19, 4.47	**0.013**	2.05	0.36, 2.50	0.053
	IV	1	1	1	1	1	1
Chemotherapy cycles							
	3	0.73	0.32, 1.65	0.451			
	4	1	1	1			
ICU admission							
	Yes	0.90	0.40, 2.02	0.796			
	No	1	1	1			
KELIM score							
	<1	1	1	1	1	1	1
	≥1	0.64	0.36, 1.16	**0.139**	0.65	0.26, 1.67	0.375
Residual disease (cm)							
	0	1	1	1	1	1	1
	<1 or ≥1	1.49	0.82, 2.71	**0.188**	0.95	0.36, 2.50	0.922
Hospital stay (days)		1.01	0.94, 1.09	0.735			

BMI: Body Mass Index, CCI: Charlson Comorbidity Index, ICU: Intensive Care Unit.

**Table 5:** Cox regression for death.

Characteristics		Univariable			Multivariable		
		OR	95% CI	*p*-Value	OR	95% CI	*p*-Value
Age (years)		1.02	0.99, 1.05	0.253			
BMI (kg/m^2^)		0.99	0.91, 1.09	0.892			
CCI		1.06	0.91, 1.25	0.454			
Clavien–Dindo classification		1.01	0.99, 1.04	0.341			
FIGO Stage							
	III	1.73	0.79, 3.79	**0.169**	0.89	0.29, 2.75	0.845
	IV	1	1	1	1	1	1
Chemotherapy cycles							
	3	0.51	0.20, 1.35	**0.176**	0.49	0.13, 1.89	0.301
	4	1	1	1	1	1	1
ICU admission							
	Yes	0.75	0.22, 2.49	0.633			
	No	1	1	1			
KELIM score							
	< 1	1	1	1	1	1	1
	≥ 1	0.43	0.21, 0.88	**0.021**	0.87	0.25, 3.01	0.862
Residual disease (cm)							
	0	1	1	1	1	1	1
	<1 or ≥1	2.98	1.44, 6.17	**0.003**	2.66	0.74.9.65	0.136
Hospital stay (days)		0.98	0.87, 1.09	0.660			

BMI: Body Mass Index, CCI: Charlson Comorbidity Index, ICU: Intensive Care Unit.

## Data Availability

In accordance with the journal’s guidelines, the data presented in this study are available on request from the corresponding author for the reproducibility of this study if such is requested.
